# Transcriptomic Profiling of *In Vitro* Tumor-Stromal Cell Paracrine Crosstalk Identifies Involvement of the Integrin Signaling Pathway in the Pathogenesis of Mesenteric Fibrosis in Human Small Intestinal Neuroendocrine Neoplasms

**DOI:** 10.3389/fonc.2021.629665

**Published:** 2021-02-24

**Authors:** Faidon-Marios Laskaratos, Ana Levi, Gert Schwach, Roswitha Pfragner, Andrew Hall, Dong Xia, Conrad von Stempel, Josephine Bretherton, Kessarin Thanapirom, Sarah Alexander, Olagunju Ogunbiyi, Jennifer Watkins, Tu Vinh Luong, Christos Toumpanakis, Dalvinder Mandair, Martyn Caplin, Krista Rombouts

**Affiliations:** ^1^ Neuroendocrine Tumour Unit, ENETS Centre of Excellence, Royal Free London NHS Foundation Trust, London, United Kingdom; ^2^ Regenerative Medicine and Fibrosis Group, Institute for Liver and Digestive Health, Royal Free Hospital, University College London, London, United Kingdom; ^3^ Otto Loewi Research Center, Medical University of Graz, Graz, Austria; ^4^ Academic Centre for Cellular Pathology, Royal Free London NHS Foundation Trust, London, United Kingdom; ^5^ Royal Veterinary College, University of London, London, United Kingdom; ^6^ Radiology Department, Royal Free London NHS Foundation Trust, London, United Kingdom; ^7^ Department of Colorectal Surgery, Royal Free London NHS Foundation Trust, London, United Kingdom

**Keywords:** neuroendocrine neoplasia, fibrosis, integrin, mesenteric desmoplasia, carcinoid

## Abstract

**Aim:**

Analysis of the pathophysiology of mesenteric fibrosis (MF) in small intestinal neuroendocrine tumors (SI-NETs) in an *in vitro* paracrine model and in human SI-NET tissue samples.

**Methods:**

An indirect co-culture model of SI-NET cells KRJ-I and P-STS with stromal cells HEK293 was designed to evaluate the paracrine effects on cell metabolic activity, gene expression by RT2 PCR Profilers to analyse cancer and fibrosis related genes, and RNA sequencing. The integrin signaling pathway, a specific Ingenuity enriched pathway, was further explored in a cohort of human SI-NET tissues by performing protein analysis and immunohistochemistry.

**Results:**

RT Profiler array analysis demonstrated several genes to be significantly up- or down-regulated in a cell specific manner as a result of the paracrine effect. This was further confirmed by employing RNA sequencing revealing multiple signaling pathways involved in carcinogenesis and fibrogenesis that were significantly affected in these cell lines. A significant upregulation in the expression of various integrin pathway – related genes was identified in the mesenteric mass of fibrotic SI-NET as confirmed by RT-qPCR and immunohistochemistry. Protein analysis demonstrated downstream activation of the MAPK and mTOR pathways in some patients with fibrotic SI-NETs.

**Conclusion:**

This study has provided the first comprehensive analysis of the crosstalk of SI-NET cells with stromal cells. A novel pathway – the integrin pathway – was identified and further validated and confirmed in a cohort of human SI-NET tissue featured by a dual role in fibrogenesis/carcinogenesis within the neoplastic fibrotic microenvironment.

## Introduction

Neuroendocrine tumors (NETs) are relatively rare cancers, but their incidence has increased dramatically over recent decades (1). NETs are often associated with the development of fibrosis, which may occur at local or distant sites (2, 3). Mesenteric fibrosis is a hallmark of small intestinal neuroendocrine tumors (SI-NETs) which has been reported to occur in up to 50% of cases (4, 5). This usually develops around a metastatic mesenteric lymph node and has a typical “spoke-wheel” appearance on imaging studies, which is considered a pathognomonic sign of these tumors (6). The development of mesenteric desmoplasia is associated with significant morbidity, and can lead to a variety of local complications, such as bowel obstruction and mesenteric ischaemia (2). More recently, its effect on overall survival has shown an association with poor prognosis (5, 7–10).

The association of NETs with fibrosis has been known for a long time. However, the pathophysiology of this relationship has not been explored in depth and the underlying mechanisms remain elusive (2). This in turn has limited our ability to develop effective antifibrotic therapies targeting specific pathways involved in this process. The lack of progress in elucidating the mechanisms of mesenteric fibrogenesis is due to the paucity of studies including functional investigations of the dynamic crosstalk between neuroendocrine tumor and stromal cells, as is shown in other tumors (11–13). The existing few studies have limitations such as the use of the pancreas derived BON-1 cell line (14) instead of an enterochromaffin cell line (15), or investigating only a small number of factors, rather than assessing entire pathways of disease (16, 17). The investigation of disease related pathways is of paramount importance in a complicated process, such as fibrosis, where multiple factors are involved, often with significant crosstalk between different pathways (2).

The present study aimed to investigate in a comprehensive manner the crosstalk of small intestinal neuroendocrine tumor cells KRJ-I (18) and P-STS (19) with HEK293 cells which were used as a stromal fibroblastic cell line (17) (20). Svedja et al. previously described a co-culture model of carcinoid-associated fibrogenesis investigating the paracrine effects between KRJ-I and HEK293 cells (17).

In this study, a new enriched pathway was identified in the crosstalk between tumor and stromal cells i.e., the integrin signaling pathway which was further validated in a cohort of human tissues derived from SI-NETs.

## Methods

### Cell Culture and Preparation of Conditioned Media

KRJ-I, P-STS (18, 19), and HEK293 cell lines were cultured in complete culture medium (1:1 dilution of Ham’s F12 and Dulbecco’s Modified Eagle Medium [DMEM] [4.5 g/l glucose] supplemented with 10% Fetal Bovine Serum [FBS] and 1% pen-strep). To obtain conditioned media, cells were cultured at a cell density of 2x10^6^ cells/T75 flask, followed by 48 h of serum starvation in culture medium with 0.5% FBS. Conditioned medium was harvested and centrifuged at 1,500 rpm for 8 min and stored at -80°C.

### Metabolic Activity Assay

Changes in metabolic activity were assessed by employing WST-1 assay (Roche). In brief, cells were cultured at a density of 25x10^3^ cells/96 well. After 24 h, cells were washed and cultured in serum free medium. Spinning steps were required at each step for KRJ-I cells, since these are cells in suspension. After a further 24 h, cells were washed and media were changed to each cell type under the following conditions: complete culture medium (10% FBS), serum free medium (0.5% FBS), KRJ-I conditioned medium, P-STS conditioned medium or HEK293 conditioned medium. 10 µl of WST-1 reagent was added per well and after 2 h of incubation changes were measured (absorbance 450/650 nm, Fluostar Omega Plate Reader (BMG labtech). Two independent experiments with four samples per condition were performed.

### RT2 Profiler Assays and Transcriptomic Analysis

KRJ-I, P-STS, and HEK293 cells were cultured at a cell density of 0.5x10^6^ cells/6 well. After 24 h, cells were washed and serum starved for 24h. This was followed by treatment for 48h under different conditions as described above: complete culture medium, serum free medium, and conditioned medium obtained from KRJ-I, P-STS, or HEK293 cells. Cells were washed, RNA was extracted by using the RNeasy mini kit (Qiagen^®^), and concentration and purity was determined using a NanoDrop 2000 Spectrophotometer (Thermo Scientific) and stored at -80°C.

Briefly, the Human Fibrosis RT Profiler PCR array was employed to investigate changes in HEK293 cells gene expression upon exposure to conditioned media of KRJ-I or P-STS cells. Three RNA samples from the same experimental condition were pooled and cDNA synthesis was based on 0.33 µg total RNA (RT2First Strand Kit, Qiagen^®^). Real-time PCR was performed in triplicate using the RT2 qPCR SYBR green mastermix and 96-well Array, and run on ABI 7500 Fast Real Time PCR System (Applied Biosystems).

The Molecular Mechanisms of Cancer RT2 Profiler PCR array was used to investigate changes in gene expression in KRJ-I and P-STS cells upon exposure to conditioned media of HEK293 cells. Samples were prepared as described above with the exception that cDNA synthesis was performed using the iScript gDNA Clear cDNA synthesis kit (Bio-Rad^®^), followed by performing the Molecular Mechanisms of Cancer RT2 Profiler PCR 96-well Array (Bio-Rad^®^) and run on ABI 7500 Fast Real Time PCR System (Applied Biosystems).

### RNA Sequencing

RNA sequencing was performed in collaboration with UCL Genomics and was performed by using three RNA samples per condition for each cell line. These samples were biological replicates.

The Library preparation of 100 ng total RNA were processed using the NEBNext RNA Ultra II kit with Poly A+ selection (p/n E7760 & E7490) according to manufacturer’s instructions. Briefly, mRNA was isolated from total RNA using paramagnetic Oligo dT beads to pull down poly-adenylated transcripts. The purified mRNA was fragmented using chemical hydrolysis (heat and divalent metal cation) and primed with random hexamers. Strand-specific first strand cDNA was generated using Reverse Transcriptase in the presence of Actinomycin D. This allowed for RNA dependent synthesis while preventing spurious DNA-dependent synthesis. The second cDNA strand was synthesised using dUTP in place of dTTP, to mark the second strand. The resultant cDNA was then “A-tailed” at the 3’ end to prevent self-ligation and adapter dimerization. Full length xGen adaptors (IDT), containing two unique 8bp sample specific indexes, a unique molecular identifier (N8) and a T overhang were ligated to the A-Tailed cDNA. Successfully ligated cDNA molecules were then enriched with limited cycle PCR (14 cycles – the actual number was dependent on the amount of input RNA). The high-fidelity polymerase employed in the PCR was unable to extend through uracil. This meant only the first strand cDNA was amplified for sequencing, making the library strand specific (first-strand).

For sequencing, the libraries to be multiplexed in the same run were pooled in equimolar quantities, calculated from Qubit and Bioanalyser fragment analysis. Samples were sequenced on the NextSeq 500 instrument (Illumina, San Diego, US) using a 75bp single read run with a corresponding 8bp unique molecular identifier (UMI) read. Samples were sequenced over 1.5 NextSeq 500 runs. The first 20 samples were combined for a full run (output of this run was 550M reads over all lanes). The second 10 samples were put on a run along with other samples that generated 480M reads. The read count table (with the precise number of reads per sample) is provided in **Supplementary File 1**. Each NextSeq run has four lanes that can only be split by indexing.

### Integrin Signaling Pathway Validation in a Cohort of Human SI-NETs

A total of 34 patients with SI-NETs, who underwent surgical resection of their primary tumor and mesenteric mass at the Royal Free Hospital, ENETS Centre of Excellence between 2016 and 2018, were prospectively recruited into this study. The study was approved by the UCL Biobank Ethical Review Committee (reference number NC2017.003). A summary of the demographic and clinical characteristics of the patients is provided in **Supplementary Table 1**. A multidimensional assessment of mesenteric fibrosis was used to classify patients into different groups of mesenteric fibrosis severity. The classification incorporates surgical, radiological (6), and histological (21, 22) parameters and provides an accurate classification of mesenteric fibrosis as we have previously described (23). Using this more precise, multidimensional evaluation of mesenteric fibrosis (see **Supplementary File 2**) we were able to classify patients into four groups: non-fibrotic, minimally fibrotic (only histological evidence of fibrosis), and mildly or severely fibrotic (depending on the amount of macroscopic fibrosis).

**Gene expression by RT-qPCR:** Briefly, snap frozen tissue samples of normal mucosa, primary tumor and mesenteric mass were homogenised using a TissueLyser II (Qiagen) and RNA was extracted using the RNeasy Mini Kit (Qiagen). RNA concentration and purity were determined using a NanoDrop 2000 spectrophotometer. Total RNA, 400 ng, was reversed transcribed using the High Capacity cDNA Reverse Transcription Kit (Thermo Fisher Scientific). RT-qPCR was run on ABI 7500 Fast Real Time PCR System using the Taqman Universal Mastermix (Thermo Fisher) with Assays-on-Demand primers (see **Supplementary Table 2**). Gene expression was normalized to normal SI mucosa (control) using GAPDH as a housekeeping gene as previously described (24).

**Immunohistochemistry:** Sections from normal SI mucosa, primary and mesenteric tumors were submitted for immunohistochemical examination using tissue samples from three patients per group (non-fibrotic, minimally fibrotic, mildly and severely fibrotic). Immunostaining was performed in paraffin-embedded sections which were de-paraffinised and hydrated through xylenes (2x, 10 min each) and ethanol (1x, 5 min 100%, 95%, 90%, 70%, 50%). Slides were either placed into 1 litre of Sodium Citrate buffer (pH 6.0) or Tris-EDTA buffer (pH 9.0) and micro-waved/pressure cooked according to the specific antibody applied (**Supplementary Table 3**). Slides were soaked in TBS with 0.04% Tween-20 (Sigma) for 5 min then blocked in peroxidase (0.3% H2O2 in methanol) for 5 min, washed in TBS for 5 min, blocked in 2.5% normal horse serum (Vector) for 10 min and then incubated overnight with primary antibodies (**Supplementary Table 3**). The slides where incubated with a biotinylated universal pan-specific secondary antibody and diaminobenzidine used as chromogen (Vector). The omission of the primary antibody was used as negative control. All sections were dehydrated, cleared in xylene, mounted with DPX (Leica biosystems), cover slipped and observed using a Zeiss Axioskop 40. Images were captured with an Axiocam IcC5 using Zeiss Axiovision (version 4.8.2) (24).

Western blot analysis was performed using tissue samples of patients from each of the mesenteric fibrosis groups (**Supplementary File 2**). Briefly, tissues (5mg/sample) were lysed in protein lysis RIPA buffer (300 µl/sample) and protein concentrations were measured with microBCA assay (ThermoFisher Scientific). During sample pooling, equal amounts of protein (8 µg/sample) and a total of 24 µg of protein was loaded in each well (n=3). Samples were separated on 4%–12% GenScript SurePAGETM gels and transferred to PVDF membranes. Membranes were incubated with blocking buffer (5% Bovine Serum Albumin [BSA]) for 1 h and incubated with primary antibodies overnight at 4°C (**Supplementary Table 3**) followed by incubation with secondary antibodies for 1 h, and protein expression was detected with SuperSignal West Pico Chemiluminescent Substrate (ThermoFisher Scientific). Band intensity was measured using Image J and GAPDH served as a loading control/housekeeping protein (24).

### Statistics

#### qRT-PCR

Statistical comparisons were performed using parametric or non-parametric tests, as appropriate, and GraphPad Prism^®^ version 8 was used for the statistical analysis. RT Profiler PCR arrays: both PCR array data passed their online quality control. For both arrays data analysis of relative gene expression was performed using the ΔΔCt method. Array analysis of the results was performed using the software provided by the Qiagen web portal (http://www.qiagen.com/geneglobe) and normalization was done on automatic HKG (housekeeping gene) panel. For the Molecular Mechanisms of Cancer RT2 Profiler PCR array analysis the Bio-rad PrimePCR Analysis software (http://www.biorad.com) was used and normalization was done on automatic HKG (housekeeping gene) panel.

#### RNA Sequence Data Analysis

Run data were demultiplexed and converted to fastq files using Illumina’s bcl2fastq Conversion Software v2.19. Fastq files were then aligned to the human genome UCSC hg38 using RNA-STAR 2.5.2b then UMI deduplicated using Je-suite (1.2.1). Reads per transcript were counted using FeatureCounts and differential expression was estimated using the BioConductor package SARTools, a DESeq2 wrapper. All annotation and sequences were obtained from Illumina iGenomes (http://emea.support.illumina.com/sequencing/sequencing_software/igenome.html). RNA sequencing data were further analysed by Ingenuity Pathway Analysis (IPA) (Qiagen IPA software). For this analysis, correction of multiple testing has been carried out using a Benjamini-Hochberg p-value adjustment. The level of controlled false positive rate was set to 0.05.

## Results

### Stromal Cells Affect the Metabolic Activity of Small Intestinal Neuroendocrine Cancer Cells

A statistically significant reduction in cell metabolic activity was observed in KRJ-I and P-STS cells when exposed to HEK293 conditioned media compared to control (serum free media) (p=0.0002) (**Supplementary Figure 1**). This is an interesting observation which is difficult to precisely relate to specific transcriptional changes investigated in this study. Many genes and pathways play different roles (often contradictory) in cell metabolism and the effect on metabolic activity ultimately depends on the crosstalk and combined effect of many different pathways. In addition, there may be several post-transcriptional and post-translational modifications that have not been assessed in our study but which could change cell metabolism.

### Gene Expression Profile Changes in Cancer Cells and Stromal Cells in a Paracrine *In Vitro* Model

**Figure 1:**
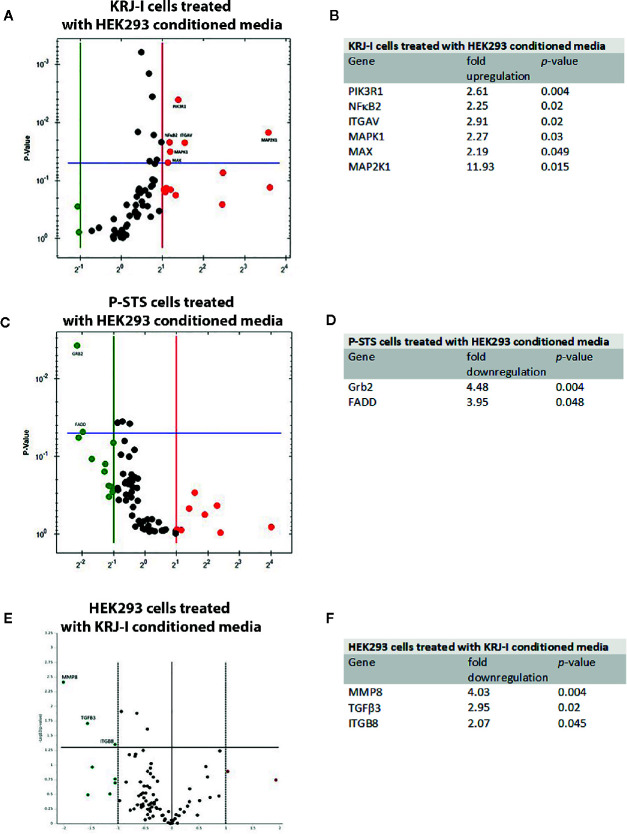
Gene expression profile changes in cancer cells and stromal cells in a paracrine *in vitro* model. Each Volcano plot shows the y-axis which represents the p-value and genes with statistically significant changes (p < 0.05). **(A)** RT Profiler “Molecular Mechanisms of Cancer” demonstrating changes in gene expression in KRJ-I cells treated with HEK293 conditioned media. **(B)** Summary of significant changes (≥2-fold, p < 0.05) in gene expression in KRJ-I cells treated with HEK293 conditioned media. **(C)** RT Profiler “Molecular Mechanisms of Cancer” demonstrating changes in gene expression in P-STS cells treated with HEK293 conditioned media. **(D)** Summary of significant changes (≥2-fold, p < 0.05) in gene expression in P-STS cells treated with HEK293 conditioned media. **(E)** “Human Fibrosis RT Profiler” demonstrating changes in gene expression in HEK293 cells treated with KRJ-I conditioned media. **(F)** Summary of significant changes (≥2-fold, p < 0.05) in gene expression in HEK293 cells treated with KRJ-I conditioned media. Results are from one experiment with three samples (n=3) per condition.

The RT2 PCR Profiler “Molecular Mechanisms of Cancer” revealed that several genes were significantly up-or down-regulated in KRJ-I cancer cells exposed to HEK293 conditioned media (fold-change≥2, p<0.05) (**Figures 1A, B** **)**. Three of these genes (MAPK1, MAP2K1 and PIK3R1) encode proteins that are components of the MAPK (mitogen-activated protein kinases) and mTOR (mammalian target of rapamycin) pathways playing a central role in the pathophysiology of carcinogenesis (25–27). The ITGAV (integrin subunit alpha v) gene encodes integrin αv which activates several signaling pathways including MAPK and mTOR and regulates a wide range of cellular processes in cancer biology (28–30). The NF-κB2 gene encodes NF-κB2/p52, a transcription factor for multiple target genes, that control many biological processes including inflammation and cell survival (31). The MAX (Myc-associated protein X) gene encodes Max, which acts as a partner for Myc regulating the expression of multiple genes (32). The Myc/Max complex targets genes involved in a wide range of biological processes (32).

On the other hand, P-STS cells treated with HEK293 media responded with a significant downregulation in Grb2 and FADD gene expression (**Figures 1C, D** **)**. The Growth factor receptor-bound protein 2 (Grb2) gene is a key adaptor protein involved in intracellular signal transduction, linking cell surface receptors to downstream signaling pathways, such as the MAPK cascade (33). Fas-associated *via* death domain (FADD) is another cytosolic adaptor protein, which is recruited to ligated death receptors, such as Fas and is characterized by pro-apoptotic features (34, 35).

To assess changes in gene expression of HEK293 cells, the “Human Fibrosis” RT PCR Profiler was employed. HEK293 cells exposed to KRJ-I conditioned media exhibited a significant downregulation of several genes (**Figures 1E, F** **)**. Metalloproteinase 8 (MMP8), also known as collagenase-2, has the ability to degrade fibrillar collagens and other substrates (36). TGFβ3 is a member of the TGFβ family which is known to be involved in fibrosis development (2) and ITGB8 is involved in the synthesis of integrin αvβ8. This integrin acts as a cell surface receptor for the latency-associated peptide (LAP) of TGFβ. Binding of latent (inactive) TGFβ to αvβ8 leads to recruitment of the metalloproteinase 14 (MMP14), which cleaves the LAP peptide and therefore activates TGFβ with induction of fibrosis (37). Finally, no significant changes were noted in gene expression of HEK293 cells treated with P-STS conditioned media (data not shown). This suggests a weak (but not absent) paracrine effect in this direction. Although there were no changes in the gene panel (84 genes) included in the “Human Fibrosis RT Profiler”, the IPA analysis showed that several transcriptional signaling pathways were significantly altered in HEK293 cells exposed to P-STS conditioned media (see next section).

### Identification of Enriched Signaling Pathways by RNA Sequencing and Ingenuity Pathway Analysis

Changes in gene expression in KRJ-I, P-STS, and HEK293 cells were further assessed with RNA sequencing followed by Ingenuity Pathway Analysis (IPA) to identify transcriptional pathways that were significantly activated or inhibited as a result of the crosstalk between cancer and stromal cells (p<0.05, |z score| ≥ 2).

**Figure 2:**
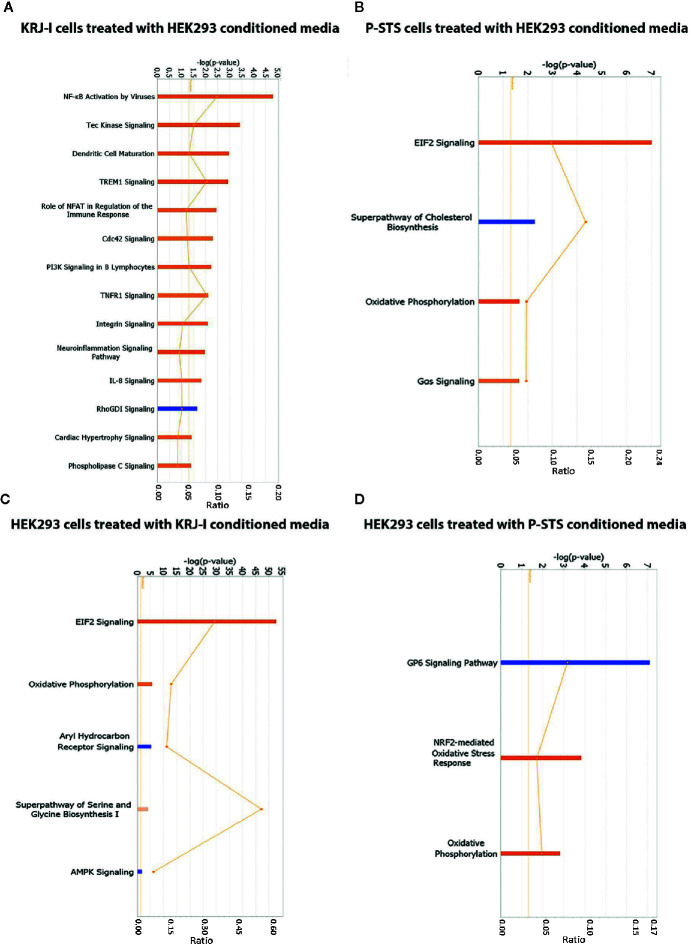
Identification of enriched signaling pathways by RNA sequencing and Ingenuity Pathway Analysis. **(A)** IPA analysis showing transcriptional pathways that are significantly altered in KRJ-I cells treated with HEK293 conditioned media. **(B)** IPA analysis showing transcriptional pathways that are significantly altered in P-STS cells treated with HEK293 conditioned media. **(C)** IPA analysis showing transcriptional pathways that are significantly altered in HEK293 cells treated with KRJ-I conditioned media. **(D)** IPA analysis showing transcriptional pathways that are significantly altered in HEK293 cells treated with P-STS conditioned media. The x-axis represents the pathways identified. Orange bars represent pathways whose transcription is activated, while blue bars represent pathways whose transcription is inhibited. The y-axis (left) shows the -log of the p-value calculated based on the Fisher’s exact test. The horizontal orange line represents the threshold of statistical significance (p < 0.05). The ratio (y-axis, right [represented by the orange points]) is calculated as follows: number of genes in a given pathway that meet cut-off criteria, divided by the total number of genes that make up that pathway.

In KRJ-I cells treated with HEK293 conditioned media the following pathways were activated: NF-KB activation by viruses, Tec kinase signaling, dendritic cell maturation, TREM1 signaling, role of NFAT in regulation of the immune response, cdc42 signaling, PI3K signaling in B lymphocytes, TNFR1 signaling, integrin signaling, neuroinflammation signaling pathway, IL-8 signaling, cardiac hypertrophy signaling, and phospholipase signaling, while the RhoGDI signaling pathway was inhibited (**Figure 2A**). Many of these pathways have been linked to oncogenesis. For example, the NF-κB activation by viruses pathway is known to be initiated by oncogenic viruses which are able to activate the NF-κB pathway *via* the canonical and non-canonical route (38). Deregulation of the NFκB pathway is known to lead to abnormal transcription of multiple target genes, involved in inflammatory and immune responses, as well as in cell survival and proliferation. As a result, activation of this pathway by oncogenic viruses is associated with the development of a wide range of malignancies (38). In addition, the role of NFAT in regulation of immune response is a pathway with oncogenic potential. NFAT proteins are transcription factors that can alter target gene expression and can affect many physiological and pathological processes, including regulation of the immune system, inflammation, angiogenesis, and development and metastasis of cancer (39). The NFAT pathway is also known to interact with other major oncogenic pathways (39). Another example is the IL-8 signaling pathway. IL-8 is a chemokine produced by many cell types that binds to its two receptors, CXCR1 (IL-8RA) and CXCR2 (IL-8RB). These receptors are then able to activate a number of signaling cascades, the most important of which are PI3K/Akt, phospholipase C/protein kinase C and Ras/Raf/ERK pathways (40). These pathways regulate critical cellular functions and ultimately lead to cell proliferation, invasion, angiogenesis and metastasis (40). IL-8 is upregulated in a wide range of malignancies due to its ability to contribute to multiple hallmarks of cancer (40), although its role in neuroendocrine neoplasia within the fibrotic stroma has not been previously reported.

In P-STS cells, treated with HEK293 conditioned media, the following pathways were significantly activated: EIF2 signaling, oxidative phosphorylation and Gas signaling, while the superpathway of cholesterol biosynthesis was inhibited (**Figure 2B**). These pathways are important for cancer growth. For example, the EIF2 signaling pathway plays a key role in global protein synthesis in eukaryotic cells and is also important in the integrated stress response by facilitating the synthesis of specific proteins that will promote cell survival under stress conditions (41). In addition, the superpathway of cholesterol biosynthesis is interesting, because lipid biogenesis is thought to play an important role in cancer growth and signaling, but to the best of our knowledge changes in lipid metabolism and their effect on disease progression have not been investigated in small bowel neuroendocrine tumors. Sterol composition of cell membranes is known to affect many membrane-based properties, such as receptor signaling and vesicular trafficking, and thus choleresterol homeostasis plays a critical role in cell growth (42).

In HEK293 cells treated with KRJ-I conditioned media, the following pathways were significantly activated: EIF2 signaling, oxidative phosphorylation, superpathway of serine and glycine biosynthesis, while the aryl hydrocarbon receptor (Ahr) and AMPK pathways were inhibited (**Figure 2C**). These pathways generally affect cell metabolism. For example, the mitochondrial oxidative phosphorylation system is involved in energy production (43) and activation of this pathway suggests an increase in cell energy production as a result of the paracrine effect. In addition, the Ahr pathway is involved in fibrogenesis and regulates the expression of genes that participate in a wide range of cellular processes, including cell growth, cell-cell adhesion, and matrix remodeling (44). The Ahr signaling pathway can alter ECM composition by regulating the expression of ECM proteins (e.g., collagen I and IV), but also the expression and activity of proteolytic enzymes, such as metalloproteinases (44). In addition, it plays an important role in cell-matrix interactions by regulating the expression of important membrane-associated proteins, such as integrins (44). It is also known to contribute to matrix remodeling by interacting with a number of other signaling pathways, such as the TGFβ and oestrogen receptor pathways (44).

In HEK293 cells treated with P-STS conditioned media, NRF2-mediated oxidative stress response and oxidative phosphorylation were significantly activated, while the GP6 signaling pathway was inhibited (**Figure 2D**). NRF2 signaling in oxidative stress is an important pathway that plays a major role in cell survival in the context of oxidative stress. The involvement of oxidative stress in carcinoid-driven fibrogenesis has been previously investigated in carcinoid heart disease and valvular fibrosis (45) but requires further evaluation in mesenteric fibrosis. In addition, the activation of oxidative phosphorylation suggests a paracrine effect on mitochondrial metabolism of stromal cells.

Hierarchical cluster analyses (**Supplementary Figure 2**) and PCA plots (**Supplementary Figure 3**) were created with representation of KRJ-I, P-STS, and HEK293 cells in different experimental conditions. In addition, a list of upstream regulators identified by IPA is shown in **Supplementary Table 4**. Although most of these upstream regulators were not differentially expressed, the overall genome wide expression profile supported the trend we observed using IPA analysis and hence biological functions. Finally, the lists of differentially expressed genes from the RNA sequencing data have been included as supplementary tables (**Supplementary Tables 5–** **12**).

### Human Fibrotic SI-NETs Are Characterized By Significant Increase in Fibrosis-Related Gene Expression and Activation of the Integrin Signaling Pathway

All *in vitro* data so far indicated that the integrin signaling pathway was significantly activated in KRJ-I cells exposed to HEK293 conditioned media. Therefore, we decided to evaluate this pathway in human tissue and investigated a set of genes known to be part of the integrin pathway (TGFβ1, COL1A1, COL3A1, fibronectin 1, ITGAV, and ITGAX). To provide a better link between *in vitro* data and human tissue data, the expression of these genes in treated KRJ-I, P-STS, and HEK293 cells in our *in vitro* co-culture model was additionally assessed and is provided in **Supplementary Table 13**.

The integrin pathway was investigated in a cohort of human tissue with SI-NETs collected prospectively from fibrotic (n=31) and non-fibrotic (n=3) patients. Therefore, in a next set of experiments we performed RT-qPCR, IHC and western blot analysis to confirm and translate these *in vitro* data in human tissues.

**Figure 3:**
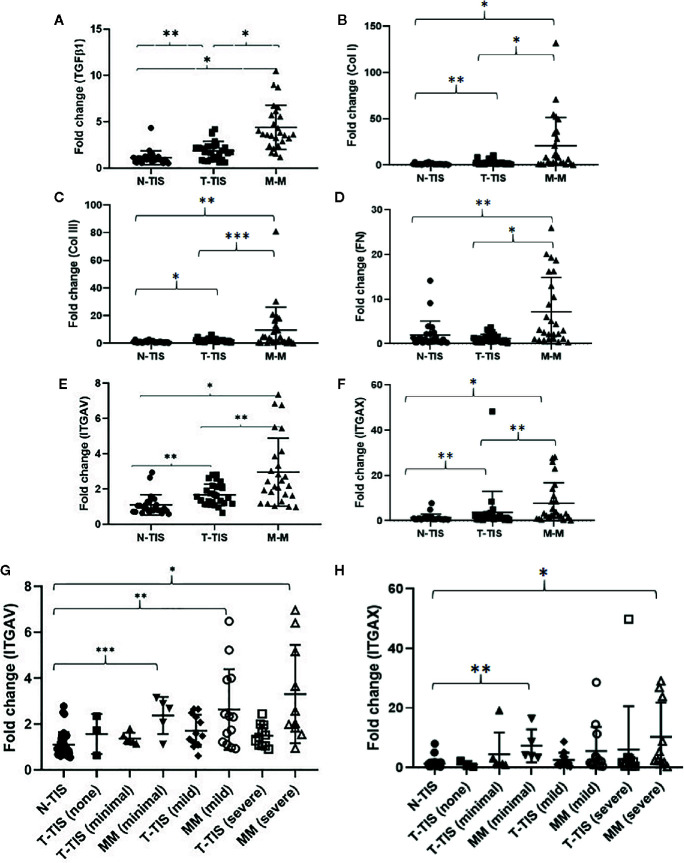
Changes in gene expression of pro-fibrogenic genes and integrins involved in the integrin signaling pathway in fibrotic SI NETs. **(A–F)** Levels of gene expression for all the evaluated genes were significantly higher in the mesenteric mass compared to the primary tumor and normal mucosa (control) (n=26 matched samples for each tissue type). Levels of gene expression for most of the evaluated genes (all except for fibronectin), were significantly higher in the primary tumor compared to normal mucosa (control) (matched samples). **(G, H)** Assessment of integrin ITGAV and ITGAX gene expression in different types of tissue across different mesenteric fibrosis patient groups revealed upregulation of these integrins in the fibrotic mesenteric mass particularly in cases with advanced fibrosis (a Dunn’s correction for multiple comparisons was performed and adjusted p-values are shown) (N-TIS [n=34), T-TIS (none) [n=3], T-TIS (minimal) [n=6], MM (minimal) [n=5], T-TIS (mild) [n=12], MM (mild) [n=13], T-TIS (severe) [n=11], MM (severe) [n=10]) *p < 0.001, **p < 0.01, ***p < 0.05. N-TIS, normal small intestinal mucosa; T-TIS, primary tumor tissue; M-M, mesenteric mass.

In non-fibrotic tumors, there were no significant differences in gene expression between the normal SI mucosa (N-TIS) and primary tumor (T-TIS) for any of the evaluated genes (TGFβ1, COL1A1, COL3A1, fibronectin 1, ITGAV, and ITGAX) (data not shown). In contrast, in fibrotic SI-NETs, there was a significantly higher expression of all the evaluated genes in the mesenteric mass (MM) compared to control normal SI mucosa (N-TIS) and primary tumor tissue (T-TIS). In addition, gene expression in the primary tumor (T-TIS) was higher compared to control (N-TIS) for most genes (TGFβ1, COL1A1, COL3A1, ITGAV, ITGAX) (matched samples) (**Figures 3A–F**). Importantly, gene expression of ITGAV and ITGAX demonstrated a significant upregulation in the fibrotic mesenteric mass in each of the different groups of mesenteric fibrosis severity (i.e., minimally fibrotic, mildly and severely fibrotic) (**Figures 3G, H**).

**Figure 4:**
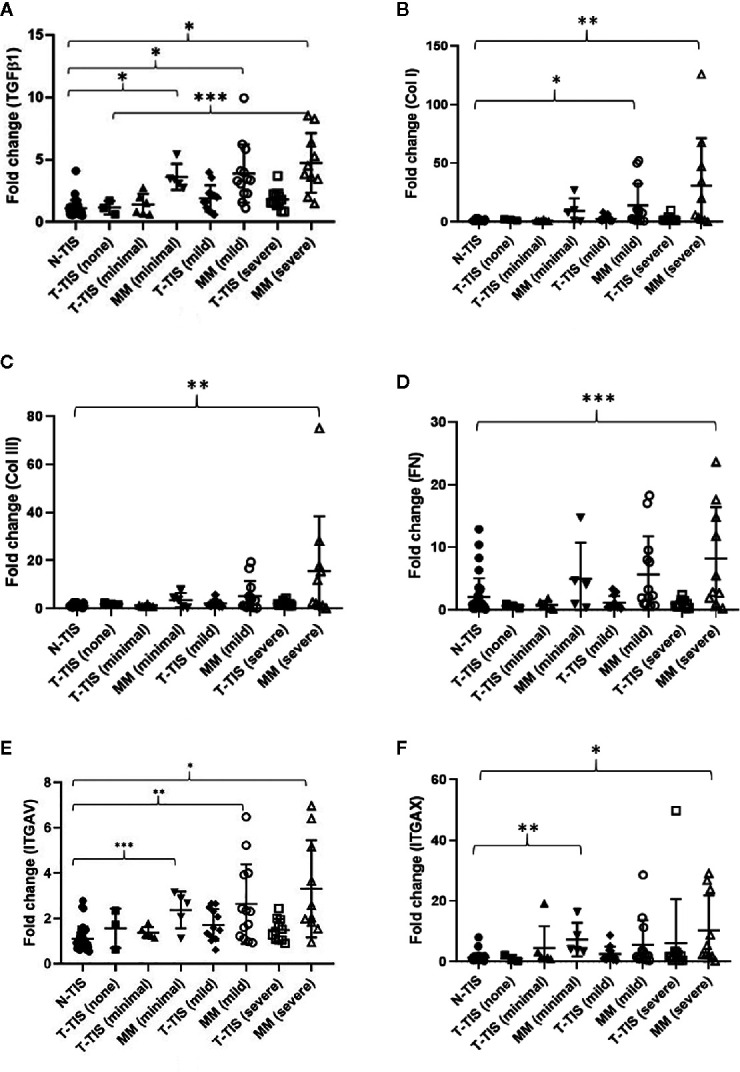
Gene expression involved in the integrin signaling pathway in SI-NETs with graded severity of mesenteric fibrosis (unmatched tumor samples compared to normal mucosa). **(A)** TGFβ1 gene expression was significantly higher in the mesenteric mass of minimally, mildly and severely fibrotic patients compared to normal SI mucosa. TGFβ1 gene expression was significantly higher in the mesenteric mass of patients with severe fibrosis compared to the primary tumor of non-fibrotic patients. **(B)** COL1A1 gene expression was significantly higher in the mesenteric mass of mildly and severely fibrotic patients compared to normal SI mucosa. **(C)** COL3A1 gene expression was significantly higher in the mesenteric mass of severely fibrotic patients compared to normal SI mucosa. **(D)** Fibronectin gene expression was significantly higher in the mesenteric mass of severely fibrotic patients compared to normal SI mucosa. **(E)** ITGAV gene expression was significantly higher in the mesenteric mass of minimally, mildly and severely fibrotic patients compared to normal SI mucosa. **(F)** ITGAX gene expression was significantly higher in the mesenteric mass of minimally and severely fibrotic patients compared to normal SI mucosa. A Dunn’s correction for multiple comparisons was performed and adjusted p-values are shown. Levels of statistical significance are indicated by asterisks: *p < 0.001, **p < 0.01, ***p < 0.05. N-TIS, normal SI mucosa; T-TIS, primary tumor tissue; MM, mesenteric mass. Number of samples in each category were, N-TIS (all patients) (n=34), T-TIS (no fibrosis) (n=3), T-TIS (minimal fibrosis) (n=6), MM (minimal fibrosis) (n=5), T-TIS (mild fibrosis) (n=12), MM (mild fibrosis) (n=13), T-TIS (severe fibrosis) (n=11), MM (severe fibrosis) (n=10).

In addition, we assessed normalized gene expression in the primary tumor (T-TIS) and mesenteric mass (MM) across different groups of mesenteric fibrosis severity (unmatched samples) (**Figure 4**). A significantly higher expression of the evaluated genes was seen in the mesenteric mass (MM) compared to normal mucosa (N-TIS), which was consistently increased, particularly in patients with severe fibrosis (**Figure 4**). This further confirms the role of the evaluated genes of the integrin signaling pathway in the neoplastic fibrotic microenvironment of SI NETs.

**Figure 5:**
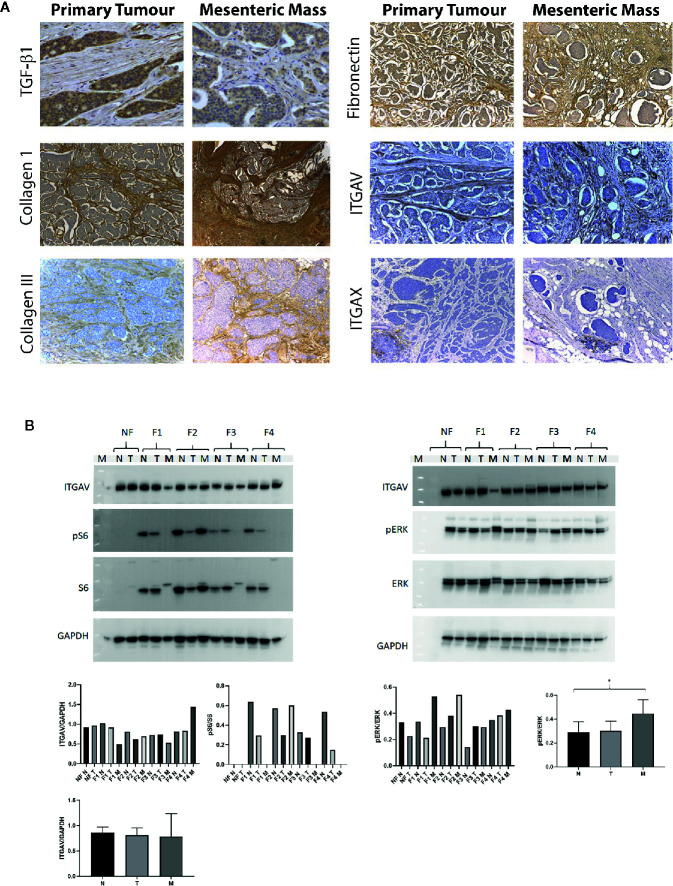
Distribution and expression of proteins involved in the integrin signaling pathway in patients with SI-NETs. **(A)** Immunohistochemistry was performed on paired primary tumor and mesenteric metastasis samples. TGFβ1 expression in the primary tumor and mesenteric mass of a mildly fibrotic midgut NET (40x magnification). Collagen I expression in the primary tumor and mesenteric mass of a patient with advanced mesenteric fibrosis (magnification 10x in primary tumor, 4x in mesenteric mass). Collagen III expression in the primary tumor and mesenteric mass of a patient with mild fibrosis (magnification 10x in both the primary and mesenteric mass). Fibronectin expression in the primary tumor and mesenteric mass of a patient with severe fibrosis (magnification 10x in both the primary tumor and mesenteric mass). ITGAV expression in the primary tumor and mesenteric mass of a patient with severe mesenteric fibrosis (magnification 20x in both the primary tumor and mesenteric mass). ITGAX expression in the primary tumor and mesenteric mass of a patient with severe mesenteric fibrosis (magnification 10x). **(B)** Representative of Western blot analysis was employed in SI-NETs tissues graded for severity of mesenteric fibrosis. Left panel, protein expression of ITGAV and downstream effectors of mTOR signaling pathway (S6, phospho-S6) in fibrotic and non-fibrotic patients with SI-NETs. Right panel, representative of Western blot analysis demonstrating the protein expression of ITGAV and downstream MAPK signaling (Erk 1/2, phospho-Erk 1/2) in fibrotic and non-fibrotic patients with SI-NETs. GAPDH served as loading control. Densitometry analyses of Western blots demonstrates ITGAV protein expression was higher in the mesenteric metastasis of patients with advanced fibrosis (F4). Heterogeneity in S6 protein expression and pS6:S6 ratio with activation within the fibrotic mesenteric mass of patients with SI-NETs. The p-Erk1/2: Erk1/2 ratio was significantly higher in the mesenteric metastasis of SI-NETs compared to normal mucosa, suggesting activation of the oncogenic MAPK pathway in the metastasis. GAPDH served as loading control. (N, normal mucosa; T. Primary tumor; M, Mesenteric mass; NF, Non-fibrotic; F1, Minimally fibrotic; F2/F3, Mildly fibrotic; F4, Severely fibrotic. N, normal mucosa; T, Primary tumor; M, Mesenteric mass). * indicates p < 0.05.

Next, the localization of these proteins, i.e., in the tumor cells, stromal cells or the ECM was further investigated by immunohistochemistry in the tissue of 12 patients (three patients per fibrosis severity group). The distribution was assessed in paired primary tumor and mesenteric metastasis samples of patients with SI-NETs and representative examples are provided in **Figure 5A**. TGFβ1 was present in the cytoplasm of both tumor cells and stromal cells whereas Collagen type I, an ECM protein, was present in the stroma with the mesenteric mass containing more stroma compared to the primary tumor. Similarly, collagen type III and fibronectin were highly expressed in the ECM. ITGAV was expressed in both tumor and stromal cells, and ITGAV expression was stronger in stromal cells compared to tumor cells. In contrast, ITGAX was expressed within inflammatory cell aggregates, but did not stain tumor and stromal cells.

Next, Western blot analysis was performed, to evaluate the protein expression of ITGAV and the activation of its downstream signaling pathways, mTOR (S6 and phospho-S6) and MAPK (Erk 1/2 and phospho-Erk 1/2). Three samples of each condition were pooled to be able to compare protein expression between the different grading conditions, i.e., F1 to F4 per membrane. We observed by densitometry a higher expression of ITGAV in the mesenteric mass of patients with advanced fibrosis (F4) relative to other tissue samples on the same blot, although there was no statistical significance in this observation due to the nature of the Western blot methodology in this experiment. Next, the presence and activation/phosphorylation of protein S6 as downstream mediator of the mTOR pathway was evaluated. Of note, no expression of S6 and P-S6 was detected in the non-fibrotic (NF) group. In contrast, significant heterogeneity was noted in the activation/phosphorylation of the S6 protein within the mesenteric metastasis of SI NETs (**Figure 5B**). Next, the MAPK signaling pathway effectors Erk1 and Erk2 and their phosphorylation profile were evaluated. The p-Erk1/2: Erk1/2 ratio was consistently higher in the mesenteric mass of patients with SI-NETs compared to normal mucosa and when samples were divided according to the three tissue categories of normal mucosa (N), primary tumor (T), and mesenteric metastasis (M), the p-Erk1/2:Erk1/2 ratio was significantly higher (p<0.05) in the metastasis group compared to control (normal mucosa) (**Figure 5B**).

## Discussion

Mesenteric fibrosis remains a poorly understood process despite its substantial associated morbidity and mortality. To our knowledge, the present study provides the first transcriptomic analysis of the crosstalk of two small intestinal neuroendocrine tumor cell lines (KRJ-I and P-STS) with stromal cells HEK293 and has delineated further the complex interactions within the tumor microenvironment. There are only a few studies in the literature that have investigated the crosstalk of neuroendocrine tumor and stromal cells and these have focused on specific, single profibrotic factors rather than pathways of disease (11, 14). Svedja et al. utilized a Transwell co-culture system of KRJ-I and HEK293 cells as a model to investigate cancer-stromal interactions within the fibrotic neoplastic microenvironment of SI NETs (17).

In the present study, the indirect co-culture system utilized two SI-NET cell lines (KRJ-I and P-STS) to investigate the complex paracrine crosstalk of neuroendocrine tumor and stromal cells. These results have revealed multiple pathway networks that are involved in both fibrogenesis and oncogenesis and suggest a bidirectional effect between cancer and stromal cells. Previous studies have focused on the effects of the tumor on fibrosis development (2), whereas the current work demonstrates that the stromal cells may lead to the activation of multiple oncogenic pathways within the cancer cells that can promote not only cancer growth but also cell invasion, angiogenesis, and metastasis. This is an important concept which may account for the epidemiological observation made recently by our group (8) and also later confirmed by others (20, 46) that the presence of mesenteric fibrosis is associated with worse overall survival and represents a feature of more aggressive tumor biology.

Based on the *in vitro* data, which showed activation of the integrin pathway in KRJ-I cells after exposure to stromal conditioned medium (**Figure 2**) as well as upregulation of several genes (ITGAV, MAPK1, MAP2K1, PIK3R1) which are involved in integrin signaling (**Figure 1**), we further investigated the integrin pathway by investigating a cohort of 34 patients with SI-NETs. According to our knowledge, this is the largest prospective study to date investigating the pathogenesis of mesenteric fibrosis in SI-NETs. Importantly, all of the investigated components of the integrin pathway were transcriptionally upregulated in the mesenteric mass. Protein analysis showed downstream activation of the MAPK oncogenic pathway in the mesenteric metastasis which could reflect a more aggressive biological behavior of cancer cells. The potentially dual role of the integrin pathway in carcinoid fibrogenesis and in neuroendocrine tumor progression induced by the fibrotic microenvironment has not been previously explored. Integrins consist of an α and β-subunit and form transmembrane proteins, which bind extracellularly to ECM components, such as fibronectin and several members of the laminin and collagen families (47). Integrins are known to transduce signals into the cell (“outside-in” signaling) *via* multiple signaling pathways, including the Ras/Raf/MEK/ERK and mTOR pathways, which were also shown to be transcriptionally active in treated KRJ-I cells (**Figure 1**). These pathways regulate a wide range of cellular processes, including cell adhesion, proliferation, survival, and migration, which contribute to cancer progression (48, 49). In addition, there is extensive crosstalk between integrins and TGFβ signaling, which is important in both cancer progression and fibrosis development (47). Although this pathway is known to contribute to cancer progression in other cancers (47, 49), its role in the mesenteric microenvironment of carcinoid tumors has not been previously investigated.

The integrin signaling pathway is known to play a role in carcinogenesis and affect the metastatic potential of many cancers and the role of integrin αv has been investigated in the context of various malignancies, such as laryngeal and hypopharyngeal squamous cell carcinomas (50), metastatic melanoma (51, 52), colorectal (53), lung (54, 55), breast (56, 57), and prostate cancer (58). ITGAV is also involved in angiogenesis, which may facilitate tumor growth independently to the direct effects of the integrin signaling pathway on cancer cell proliferation (59). Furthermore, ITGAV is known to play an important role in fibrosis development in many chronic fibrotic conditions and some malignancies, mainly through regulation of TGFβ activity in the ECM, and a variety of inhibitors have been evaluated in animal models (60). Several small molecule inhibitors (such as cilengitide) and function-blocking monoclonal antibodies have been developed and have shown promising results as antifibrotic agents in this context (60).

Although our study has provided insight into the complex mechanisms of mesenteric fibrogenesis in SI-NETs, it has several limitations. Firstly, neuroendocrine cell lines have many known limitations (61). For example, even though the KRJ-I cell line has been previously well characterized and defined as a small intestinal neuroendocrine cell line (15), Hofving et al. have recently challenged its authenticity and concluded that it may be of lymphoblastoid origin (62, 63). Similarly, we chose to use HEK293 cells as a stromal cell line based on the previous literature on carcinoid-related fibrogenesis that utilized these cells as a fibroblastic cell line (17, 20). Secondly, our *in vitro* model was designed to evaluate paracrine effects, while the mesenteric tumor microenvironment is more complex and includes direct communication between many different cell types, but also the effects of cell adhesion on extracellular matrix, which may be important in the pathophysiology of fibrosis (2, 64). Therefore, we opted to validate these *in vitro* data in a prospective cohort of human Si-NETs which was well characterized and classified (23). Using this cohort, we showed a strong significance at the transcriptional level whereas limitations were detectable at the protein levels, suggesting that more patients should be recruited in the near future.

In conclusion, the mesenteric fibrotic microenvironment is a nidus of complex interactions that promote both carcinogenesis and fibrogenesis. These mechanisms need to be evaluated in a systematic way, in order to better understand the underlying pathophysiology and develop effective antifibrotic and antineoplastic drugs.

## Data Availability Statement

All datasets generated for this study are included in the **Supplementary Files**.

## Ethics Statement

The studies involving human participants were reviewed and approved by UCL Biobank Ethical Review Committee. The patients/participants provided their written informed consent to participate in this study.

## Author Contributions

Conceptualization: F-ML, MC, and KR. Methodology and investigation: F-ML, AL, GS, RP, AH, DX, CV, JB, KT, SA, OO, JW, and TL. Validation: KR, F-ML, and AL. Formal analysis: DX. Data curation: F-ML.Writing—original draft: F-ML and KR. Visualization: F-ML and KR. Writing—review and editing: F-ML, MC, and KR. Resources: CT and DM. Funding acquisition: MC. Supervision: MC and KR. Project administration: KR. All authors contributed to the article and approved the submitted version.

## Funding

This work was supported by the Royal Free Charity.

## Conflict of Interest

The authors declare that the research was conducted in the absence of any commercial or financial relationships that could be construed as a potential conflict of interest.

## Supplementary Material

Supplementary File 1 Read count table summarizing number of reads for each sample analysed by RNA sequencing.

Supplementary Figure 1 Changes in cell metabolic activity observed in the SI-NET cell lines KRJ-I and P-STS in different experimental conditions. Changes in metabolic activity were assessed using the WST-1 assay. Absorbance was read at 450/650nm and measured 2h after adding the WST-1 reagent. A statistically significant reduction in cell metabolic activity was seen in KRJ-I and P-STS cells treated with HEK293 conditioned media compared to control (serum free media) (*p=0.0002). Results are from 2 independent experiments (n= 8 samples per condition). CM: complete media, SFM: serum free media, HCM: HEK293 conditioned media.

Supplementary Figure 2 Cluster dendrograms for KRJ-I, P-STS and HEK293 cells in different experimental conditions.

Supplementary Figure 3 PCA (Principal Component Analysis) plots for KRJ-I, P-STS and HEK293 cells.

Supplementary Table 5 Differentially expressed genes (downregulated) in KRJ-I cells using RNA sequencing analysis.

Supplementary Table 6 Differentially expressed genes (upregulated) in KRJ-I cells using RNA sequencing analysis.

Supplementary Table 7 Differentially expressed genes (downregulated) in P-STS cells using RNA sequencing analysis.

Supplementary Table 8 Differentially expressed genes (upregulated) in P-STS cells using RNA sequencing analysis.

Supplementary Table 9 Differentially expressed genes (downregulated) in HEK293 cells exposed to KRJ-I conditioned media using RNA sequencing analysis.

Supplementary Table 10 Differentially expressed genes (upregulated) in HEK293 cells exposed to KRJ-I conditioned media using RNA sequencing analysis.

Supplementary Table 11 Differentially expressed genes (upregulated) in HEK293 cells exposed to P-STS conditioned media using RNA sequencing analysis.

Supplementary Table 12 Differentially expressed genes (upregulated) in HEK293 cells exposed to P-STS conditioned media using RNA sequencing analysis.
