# Supplementary material for: Transcriptomic Profiling of In Vitro Tumor-Stromal Cell Paracrine Crosstalk Identifies Involvement of the Integrin Signaling Pathway in the Pathogenesis of Mesenteric Fibrosis in Human Small Intestinal Neuroendocrine Neoplasms
Source: Front Oncol. 2021 Feb 24;11:629665. doi: 10.3389/fonc.2021.629665 (PMC7943728; doi:10.3389/fonc.2021.629665)
Supplement: Supplementary file 2 [file DataSheet_2.docx]

**Supplementary file 2. Multidimensional assessment of mesenteric fibrosis used to classify patients into different groups of mesenteric fibrosis severity**

A multidimensional assessment of mesenteric fibrosis was used to accurately classify the mesenteric fibrosis severity, which incorporated surgical, radiological and histological criteria, that we have previously described [25]. The following components of this assessment were assessed:

**i).** The radiological severity of mesenteric desmoplasia was based on the scoring system originally proposed by Pantongrag-Brown et al [6] using the following categories: a. No radiological evidence of mesenteric desmoplasia (Absence of radiating strands), b. Mild desmoplasia (≤10 thin radiating strands), c. Moderate desmoplasia (>10 thin strands or <10 thick strands) and d. Severe desmoplasia (≥10 thick strands)

**ii).** The histological assessment of mesenteric fibrosis was based on the histological slide with the maximum amount of fibrous tissue. The histological slide was stained with a connective tissue stain (Sirius Red) and two parameters were measured:

**a).** The width of the thickest fibrous band surrounding the mesenteric mass. This technique was used previously by Pantongrag-Brown et al and showed a correlation with the radiological assessment of mesenteric fibrosis [6].

**b).** The Collagen Proportionate Area (CPA), which represents the percentage of collagen in the stroma surrounding the mesenteric metastatic tumour. This is a quantitative method of measuring fibrous tissue using digital image analysis and has been validated in liver cirrhosis [23, 24].

**Optimisation/characterisation of the inter-observer variability**

The cross-sectional imaging (CT/MRI scan) was assessed independently by 2 assessors (CS, JB) with good inter-observer agreement. In a small number of cases (n=3) a minor discrepancy was observed between the two assessments and consensus was reached between the assessors to determine the radiological severity of the mesenteric desmoplasia after a final review of the imaging studies.

The histology (mesenteric mass with surrounding fibrosis) were assessed independently by 2 assessors (AH, SA) with good inter-observer variability (CPA r=0.86998 [95% CI 0.7487, 0.9347], p<0.0001; width of fibrous bands r=0.9174 [95% CI 0.8366, 0.9591], p<0.0001). In the case of minor discrepancies (<20% difference between the two measurements) the mean value of the two assessments was calculated and used for our analysis. In the small number of cases with more significant discrepancies (>20% difference between the two measurements), consensus was reached between the two assessors regarding the CPA and width of fibrous band measurements after a final review of the histology slides.

**iii).** A surgical (intra-operative) assessment of the extent of mesenteric fibrosis in relation to the entire small bowel mesentery was also provided using the following categories: a. No desmoplasia (No mesenteric fibrosis), b. Mild desmoplasia (Mesenteric fibrosis involving <25% of the small bowel mesentery), c. Moderate desmoplasia (Mesenteric fibrosis involving 25-50% of the small bowel mesentery) and d. Severe desmoplasia (Mesenteric fibrosis involving >50% of the small bowel mesentery). This assessment was provided by the operating surgeon (the same surgeon [OO] performed the macroscopic assessment of mesenteric desmoplasia in all the cases).

***Optimal cut-off points of histological parameters (maximum width and CPA) for the prediction of clinical fibrosis***

A receiver operating characteristic (ROC) curve was used to establish the optimal cut-off points of CPA and maximum width of fibrous bands that predicted with the best sensitivity and specificity the presence of clinical (radiological and/or surgical) fibrosis.

A width of fibrous band > 0.505mm and a CPA >37.6% were identified as the optimal cut-off points (Width of fibrous bands: AUC 0.751 [95% CI 0.535, 0.967], p=0.027 and CPA (AUC 0.804 [95% CI 0.634, 0.975], p=0.007).

**Development of different patient groups of mesenteric fibrosis severity**

A total of 34 patients were grouped in different categories of mesenteric fibrosis severity:

**1).** Non-fibrotic group: No evidence of mesenteric fibrosis (n=3)

**2).** Minimally fibrotic group: Only histological (but no clinical evidence of) fibrosis (n=6). In this group of patients, a mesenteric metastasis was present and surrounded by a small amount of fibrosis detected only histologically.

**3).** Mildly and severely fibrotic groups: These patients had evidence of clinical (macroscopic) fibrosis (n=25). The group of patients with clinical fibrosis was further sub-divided into 2 smaller subgroups: A group of patients with mild fibrosis (n=14) and another group with severe fibrosis (n=11). A scoring system was developed to group patients in different categories of clinical fibrosis severity. This scoring system (**Table**) incorporated surgical (macroscopic), radiological and histological parameters.

**Table. Scoring system used to assess the severity of mesenteric fibrosis. This system is not validated, but incorporates surgical, radiological and histological measurements, therefore allowing for a more objective, multidimensional assessment of the severity of mesenteric fibrosis [25]. CPA: Collagen Proportionate Area.**

| Surgical evidence of fibrosis | Radiological evidence of fibrosis | Histological evidence of fibrosis |
| --- | --- | --- |
| 0: None | 0: None | 0: None |
| 1: <25% of small bowel mesentery | 1: ≤10 thin strands | 1: Yes, but CPA<37.6% AND width of fibrous band <0.505 mm |
| 2: 25-50% of small bowel mesentery | 2: >10 thin and <10 thick strands | 2: Yes, CPA>37.6% OR width of fibrous band >0.505 mm |
| 3: >50% of small bowel mesentery | 3: ≥10 thick strands | 3: Yes, CPA>37.6% AND max width >0.505mm |

A total score of 6 was arbitrarily chosen as a cut-off point to allow a fairly equal distribution of patients in the two subgroups of clinical fibrosis (mild <6, severe ≥6).

Interestingly, the non-fibrotic group was characterised by the absence of a mesenteric mass, while all patients with a mesenteric metastasis had evidence of fibrosis development, and the extent of the desmoplastic reaction varied significantly, from minimal (detected only histologically) to more advanced.

Using this methodology, patients were grouped into 4 distinct categories with graded severity of mesenteric fibrosis, i.e. patients with no mesenteric fibrosis (n=3), minimal fibrosis (microscopic fibrotic capsule around the mesenteric lymph node) (n=6), mild (n=14) and severe mesenteric fibrosis (n=11).
