# Supplementary figures and images for: Transcriptomic Profiling of In Vitro Tumor-Stromal Cell Paracrine Crosstalk Identifies Involvement of the Integrin Signaling Pathway in the Pathogenesis of Mesenteric Fibrosis in Human Small Intestinal Neuroendocrine Neoplasms
Source: Front Oncol. 2021 Feb 24;11:629665. doi: 10.3389/fonc.2021.629665 (PMC7943728; doi:10.3389/fonc.2021.629665)

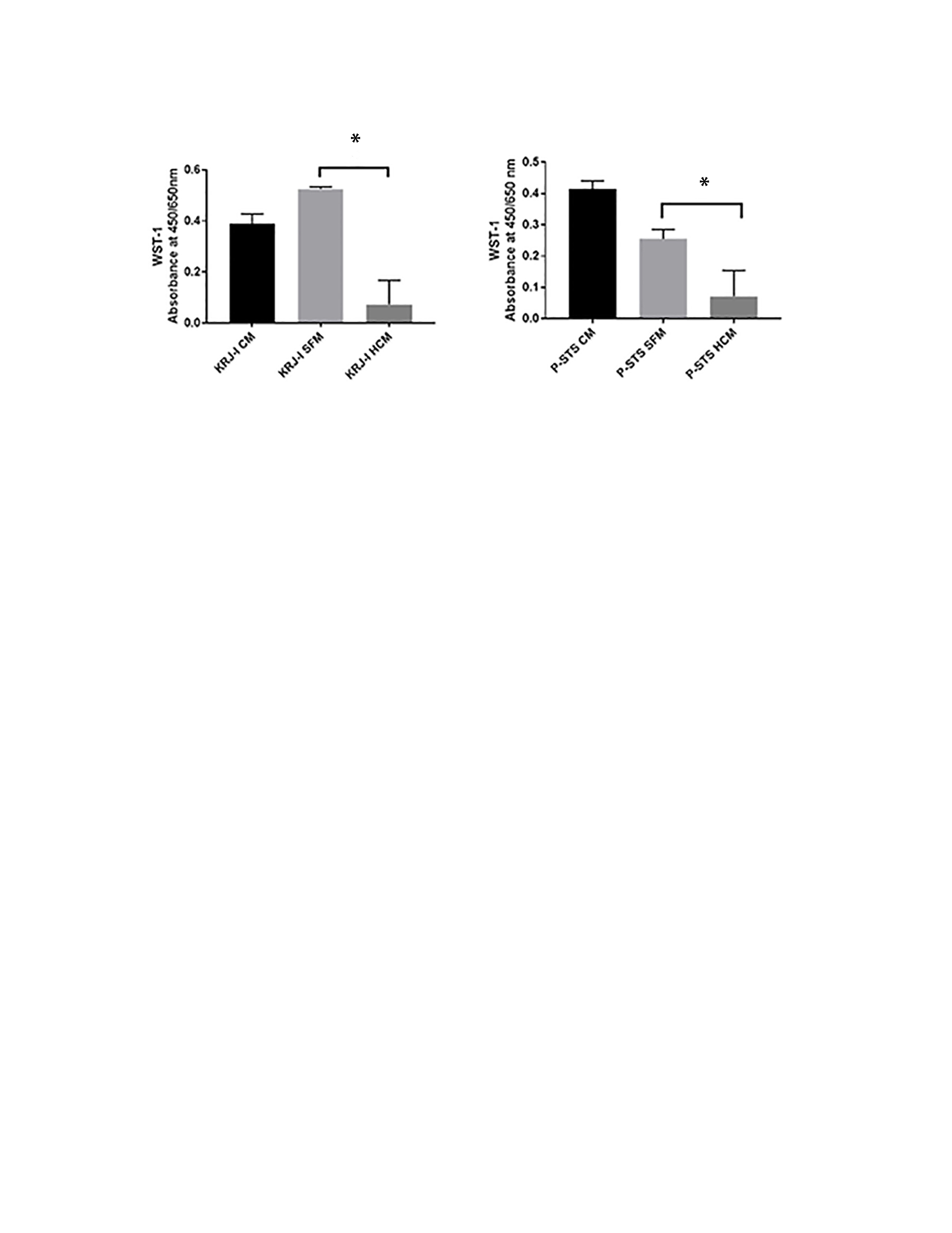

Supplement: Supplementary Figure 1 — Changes in cell metabolic activity observed in the SI-NET cell lines KRJ-I and P-STS in different experimental conditions. Changes in metabolic activity were assessed using the WST-1 assay. Absorbance was read at 450/650nm and measured 2h after adding the WST-1 reagent. A statistically significant reduction in cell metabolic activity was seen in KRJ-I and P-STS cells treated with HEK293 conditioned media compared to control (serum free media) (*p=0.0002). Results are from 2 independent experiments (n= 8 samples per condition). CM: complete media, SFM: serum free media, HCM: HEK293 conditioned media. [file Image_1.tif]

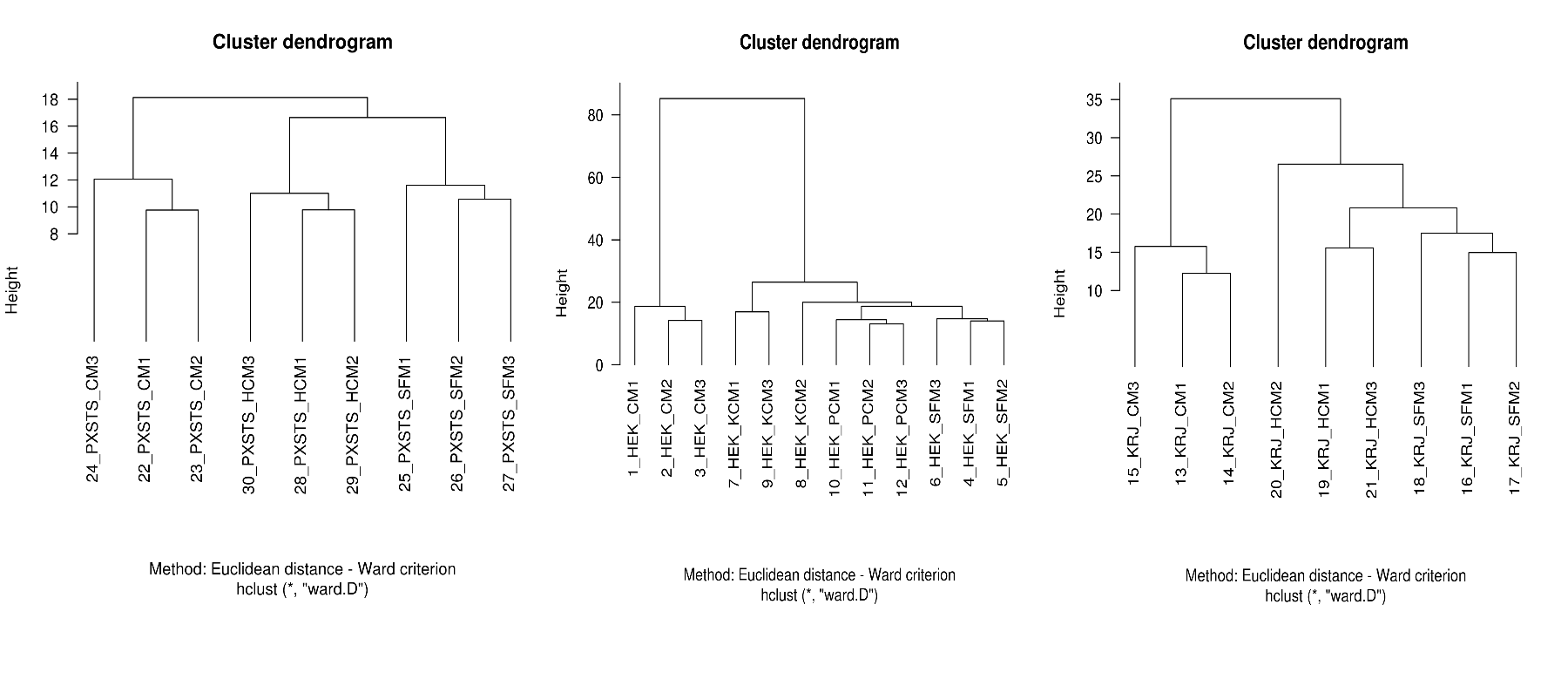

Supplement: Supplementary Figure 2 — Cluster dendrograms for KRJ-I, P-STS and HEK293 cells in different experimental conditions. [file Image_2.tiff]

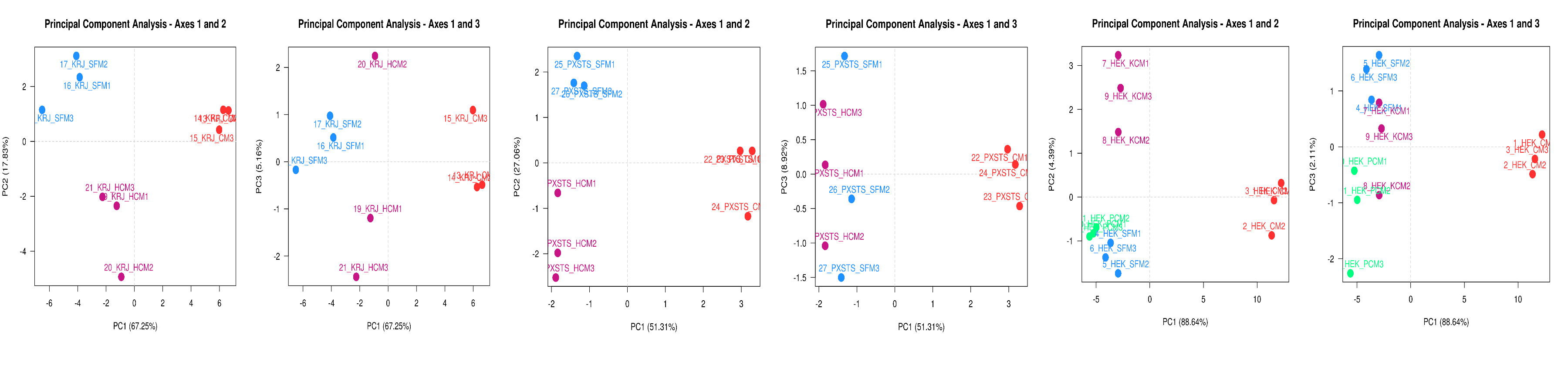

Supplement: Supplementary Figure 3 — PCA (Principal Component Analysis) plots for KRJ-I, P-STS and HEK293 cells. [file Image_3.tiff]
