# Supplementary material for: Transcriptomic Profiling of In Vitro Tumor-Stromal Cell Paracrine Crosstalk Identifies Involvement of the Integrin Signaling Pathway in the Pathogenesis of Mesenteric Fibrosis in Human Small Intestinal Neuroendocrine Neoplasms
Source: Front Oncol. 2021 Feb 24;11:629665. doi: 10.3389/fonc.2021.629665 (PMC7943728; doi:10.3389/fonc.2021.629665)
Supplement: Supplementary file 6 [file Table_1.docx]

**Table S1. Summary of demographic and clinical characteristics of patients enrolled in the study**

| Demographic and clinical characteristics | Patients with midgut NETs who underwent surgery (n=34) n (%) |
| --- | --- |
| Age (mean±SD) | 61±13 |
| Sex  Male  Female | 23 (68%)  11 (32%) |
| Grade  1  2 | 21 (62%)  13 (38%) |
| Extent of disease  Localised  Locoregional  Metastatic | 3 (9%)  9 (26%)  22 (65%) |
| Mesenteric mass | 31 (91%) |
| Liver metastases | 17 (50%) |
| Distant extrahepatic metastases | 10 (29%) |
| Mesenteric fibrosis | 31 (91%) |
| Medical therapy  Octreotide LAR  Lanreotide Autogel | 10 (29%)  8 (24%) |
| Surgical therapy  Small bowel resection  Right hemicolectomy (R0)  Right hemicolectomy (R1) | 1 (3%)  24 (71%)  9 (26%) |
