# Supplementary material for: Transcriptomic Profiling of In Vitro Tumor-Stromal Cell Paracrine Crosstalk Identifies Involvement of the Integrin Signaling Pathway in the Pathogenesis of Mesenteric Fibrosis in Human Small Intestinal Neuroendocrine Neoplasms
Source: Front Oncol. 2021 Feb 24;11:629665. doi: 10.3389/fonc.2021.629665 (PMC7943728; doi:10.3389/fonc.2021.629665)
Supplement: Supplementary file 7 [file Table_2.docx]

**Table S2**. Assays-on-demand primers used for RT-qPCR in human tissue samples of patients with fibrotic and non-fibrotic SI NETs.

| Gene | Assay-on-demand primer |
| --- | --- |
| COL1A1 | Hs00164004 |
| COL3A1 | Hs00943809 |
| FN1 | Hs01549976 |
| TGFβ1 | Hs00998133 |
| ITGAV | Hs00233808 |
| ITGAX | Hs00174217 |
| GAPDH | Hs02786624 |
