# Supplementary material for: Transcriptomic Profiling of In Vitro Tumor-Stromal Cell Paracrine Crosstalk Identifies Involvement of the Integrin Signaling Pathway in the Pathogenesis of Mesenteric Fibrosis in Human Small Intestinal Neuroendocrine Neoplasms
Source: Front Oncol. 2021 Feb 24;11:629665. doi: 10.3389/fonc.2021.629665 (PMC7943728; doi:10.3389/fonc.2021.629665)
Supplement: Supplementary file 8 [file Table_3.docx]

**Table S3.** **Summary of primary antibodies, pre-treatments and dilutions used for immunohistochemistry.**

| **Primary antibody** | **Host** | **Company** | **Product code** | **Pre-treatment** | **Dilution** |
| --- | --- | --- | --- | --- | --- |
| Collagen I | Rabbit pAb | AbCam | ab34710 | Trypsinisation for 30 mins | 1:200 |
| Collagen III | Rabbit pAb | AbCam | ab7778 | Citrate buffer and microwave for 20 mins | 1:300 |
| Fibronectin | Rabbit pAb | Abcam | ab2413 | Citrate buffer and microwave for 10 mins | 1:200 |
| CD11c  (Integrin alpha-X/beta-2) | Rabbit pAb | Abcam | ab52632 | Tris-EDTA and microwave for 10 mins | 1:500 |
| Integrin alpha v | Rabbit pAb | Abcam | ab179475 | Citrate buffer and microwave for 20 mins | 1:250 |
| TGFβ1 | Rabbit pAb | Abcam | ab92486 | Citrate buffer and microwave for 20 mins | 1:75 |
