# Supplementary material for: Transcriptomic Profiling of In Vitro Tumor-Stromal Cell Paracrine Crosstalk Identifies Involvement of the Integrin Signaling Pathway in the Pathogenesis of Mesenteric Fibrosis in Human Small Intestinal Neuroendocrine Neoplasms
Source: Front Oncol. 2021 Feb 24;11:629665. doi: 10.3389/fonc.2021.629665 (PMC7943728; doi:10.3389/fonc.2021.629665)
Supplement: Supplementary file 9 [file Table_4.docx]

| **Cell line (comparison of treated vs. untreated cells)** | **Top upstream regulator** | **p-value of overlap** | **Predicted activation** | **Differentially expressed** | **Direction of regulation** | **Adj p-value** |
| --- | --- | --- | --- | --- | --- | --- |
| KRJ-I (HCM vs SFM) | Lipopolysaccharide  TNF  TP53  IFNG  IL4 | 2.00E-17  2.33E-14  3.94E-13  6.32E-13  2.00E-12 | Activated  Activated  Activated  Activated  Activated | No  No  No  No  No |  |  |
| P-STS (HCM vs SFM) | Sirolimus  ST1926  MYCN  5-fluorouracil  CD 437 | 2.00E-09  3.91E-08  9.18E-08  2.93E-07  6.29E-06 | Inhibited  Inhibited  Activated  Inhibited  Inhibited | No  No  No  No  No |  |  |
| HEK293 (KCM vs SFM) | MYCN  Sirolimus  **RICTOR**  5-fluorouracil  CD 437 | 1.04E-44  3.35E-31  **4.26E-28**  6.27E-22  7.80E-22 | Activated  Inhibited  **Inhibited**  Inhibited  Inhibited | No  No  **Yes**  No  No | **Down** | **0.037** |
| HEK293 (PCM vs SFM) | ST1926  CD 437  NFE2L2  1,2-dithiol-3-thione  COLQ | 2.27E-12  5.68E-12  2.82E-08  8.20E-08  1.05E-06 | Inhibited  Inhibited  Activated  Activated | No  No  No  No  No |  |  |

**Table S4. List of upstream regulators identified by IPA analysis in KRJ-I, P-STS and HEK293 cells and connection with expression data**

*HCM: HEK293 conditioned media, SFM: Serum free media, KCM: KRJ-I conditioned media, PCM: P-STS conditioned media
