# Supplementary material for: Transcriptomic Profiling of In Vitro Tumor-Stromal Cell Paracrine Crosstalk Identifies Involvement of the Integrin Signaling Pathway in the Pathogenesis of Mesenteric Fibrosis in Human Small Intestinal Neuroendocrine Neoplasms
Source: Front Oncol. 2021 Feb 24;11:629665. doi: 10.3389/fonc.2021.629665 (PMC7943728; doi:10.3389/fonc.2021.629665)
Supplement: Supplementary file 18 [file Table_13.docx]

|  | **KRJ-I** | | **P-STS** | | **HEK (KCM)** | | **HEK (PCM)** | |
| --- | --- | --- | --- | --- | --- | --- | --- | --- |
| **Gene** | **Fold change** | **Adjusted p-value** | **Fold change** | **Adjusted p-value** | **Fold change** | **Adjusted p-value** | **Fold change** | **Adjusted p-value** |
| COL1A1 | 1.293 | 0.328 | 1.28 | 0.226 | 1.208 | 0.068 | 0.897 | 0.213 |
| COL3A1 | No expression |  | No expression |  | **2.089** | **<0.0001** | **0.705** | **0.0002** |
| TGFβ1 | 1.108 | 0.315 | 0.936 | 0.756 | **0.65** | **<0.0001** | 0.956 | 0.539 |
| FN-1 | No expression |  | 0.892 | 0.727 | **2.869** | **<0.0001** | 0.808 | 0.073 |
| ITGAV | **2.91**** | **0.02**** | -1.24** | 0.799** | **0.853** | **0.002** | 0.988 | 0.926 |
| ITGAX | **2.138** | **<0.0001** | No expression |  | 1.421 | 0.630 | 0.361 | 0.101 |

**Table S13**. Assessment of changes in gene expression in integrin pathway genes in cell lines KRJ-I, P-STS, HEK293.

*Results were based on RNA sequencing data except for cases where gene changes had been assessed using RT Profiler data

**RT profiler data
